# Comparative Transcriptomics of the Bovine Apicomplexan Parasite *Theileria parva* Developmental Stages Reveals Massive Gene Expression Variation and Potential Vaccine Antigens

**DOI:** 10.3389/fvets.2020.00287

**Published:** 2020-06-09

**Authors:** Kodzo Atchou, Juliette Ongus, Eunice Machuka, John Juma, Christian Tiambo, Appolinaire Djikeng, Joana C. Silva, Roger Pelle

**Affiliations:** ^1^Institute for Basic Sciences, Technology and Innovation, Pan African University, Nairobi, Kenya; ^2^Biosciences eastern and central Africa—International Livestock Research Institute (BecA-ILRI), Nairobi, Kenya; ^3^Centre for Tropical Livestock Genetics and Health, The Roslin Institute and Royal (Dick) School of Veterinary Studies, The University of Edinburgh, Scotland, United Kingdom; ^4^Institute for Genome Sciences, University of Maryland School of Medicine, Baltimore, MD, United States; ^5^Department of Microbiology and Immunology, University of Maryland School of Medicine, Baltimore, MD, United States

**Keywords:** *Theileria parva*, schizont, piroplasm, transcriptome, vaccine antigens

## Abstract

*Theileria parva* is a protozoan parasite that causes East Coast fever (ECF), an economically important disease of cattle in Africa. It is transmitted mainly by the tick *Rhipicephalus appendiculatus*. Research efforts to develop a subunit vaccine based on parasite neutralizing antibodies and cytotoxic T-lymphocytes have met with limited success. The molecular mechanisms underlying *T. parva* life cycle stages in the tick vector and bovine host are poorly understood, thus limiting progress toward an effective and efficient control of ECF. Transcriptomics has been used to identify candidate vaccine antigens or markers associated with virulence and disease pathology. Therefore, characterization of gene expression throughout the parasite's life cycle should shed light on host–pathogen interactions in ECF and identify genes underlying differences in parasite stages as well as potential, novel therapeutic targets. Recently, the first gene expression profiling of *T. parva* was conducted for the sporoblast, sporozoite, and schizont stages. The sporozoite is infective to cattle, whereas the schizont is the major pathogenic form of the parasite. The schizont can differentiate into piroplasm, which is infective to the tick vector. The present study was designed to extend the *T. parva* gene expression profiling to the piroplasm stage with reference to the schizont. Pairwise comparison revealed that 3,279 of a possible 4,084 protein coding genes were differentially expressed, with 1,623 (49%) genes upregulated and 1,656 (51%) downregulated in the piroplasm relative to the schizont. In addition, over 200 genes were stage-specific. In general, there were more molecular functions, biological processes, subcellular localizations, and pathways significantly enriched in the piroplasm than in the schizont. Using known antigens as benchmarks, we identified several new potential vaccine antigens, including TP04_0076 and TP04_0640, which were highly immunogenic in naturally *T. parva*-infected cattle. All the candidate vaccine antigens identified have yet to be investigated for their capacity to induce protective immune response against ECF.

## Introduction

Cattle production constitutes a significant component of agriculture, economy, and food security in the world, especially in developing countries (1, 2). East Coast fever (ECF) is a lymphoproliferative and lymphodestructive disease of cattle caused by the hemoprotozoan *Theileria parva*, mainly transmitted by the tick vector *Rhipicephalus appendiculatus*. ECF kills one cow every 30 s and has a devastating impact on pastoralists and smallholder farmers because of its rapid effect, since animals often die within 3 to 4 weeks of infection [reviewed by (3)]. This causes significant economic losses in 12 countries in eastern, central, and southern Africa regions (3–6). As the infected tick feeds on cattle, the sporozoites are inoculated in the mammalian host at the feeding site. The sporozoites then invade host lymphocytes and differentiate into multinucleate bodies, called schizonts, in the cytoplasm of infected lymphocytes after a period of 3 days. Schizonts cause the transformation of infected host white blood cells, inducing a phenotype similar to cancer (7, 8). Schizonts undergo merogony, and the released merozoites invade erythrocytes and form piroplasms, which are infective to the feeding tick that ingests parasitized erythrocytes (9). A method of vaccination, whereby infection of cattle with live *T. parva* sporozoites is done simultaneously with treatment with long-acting oxytetracycline, was developed over 40 years ago (10). This resulted in a live vaccine called the Muguga Cocktail, which was developed based on a combination of three *T. parva* stocks, the Muguga, Kiambu 5, and Serengeti-transformed stocks. The Muguga Cocktail generates long-lasting immunity in vaccinated cattle against challenge with homologous *T. parva* stocks. However, ITM-vaccinated animals usually remain carriers of the vaccine parasite strains and a source of infections to ticks. Tremendous progress was made on ECF research. Publication of a reference genome sequence of *T. parva* has led to a more thorough characterization of the pathogen and of the Muguga Cocktail strains (11–13). But, extensive efforts to develop alternative, more easily manufactured and user-friendly, subunit vaccines have met with limited successes (3). Therefore, it is imperative to identify more candidate vaccine antigens. Gene expression profiling, including high-throughput transcriptomics, has been used to identify potential diagnostic and therapeutic targets as well as to correlate gene expression profiles to pathologic diagnosis, clinical outcomes, or therapeutic response (14, 15). Moreover, transcriptomics enables predictive analysis of the structure, location, role, and functional motifs of genes and its product. Recently, a comparative transcriptome profiling of *T. parva* was done on two life cycle stages in ticks, the sporoblast and the sporozoite (the latter is transmissible from tick to cow upon tick feeding), and on the pathogenic schizont stage (16). No work has yet been done on the piroplasm stage that is transmissible from cattle to ticks. That first comparative transcriptomic analysis revealed that the development of the parasite from the sporozoite in the tick vector into the schizont in the bovine host cells is accompanied by a drastic increase of upregulated genes, though the 10 most highly expressed genes occurred in the arthropod stages. It also identified several genes with expression similar to known candidate vaccine antigen genes and revealed errors in the structural annotation of the *T. parva* genome. The present study was then set up to extend the analysis of *T. parva* gene expression profiles to the piroplasm stage in comparison to the schizont stage, using the Illumina MiSeq next-generation sequencing platform. Furthermore, data from previous (16) and current studies were combined in our analysis for the search of new candidate vaccine antigens.

## Materials and Methods

### Sample Collection and Purification

#### Ethics Statements

The study reported here was carried out in strict accordance with the recommendations in the standard operating procedures of the ILRI IACUC and adequate consideration of the 3R's (replacement of animal with non-animal techniques, reduction in the number of animals used, and refinement of techniques and procedures that reduce pain and distress). The ILRI's Experimental Animal Request Form and Protocol for blood collection was approved by the ILRI IACUC (IACUC ref no. 2006.9, IACUC ref no. 2006.10, IACUC ref no. 2007.10, and IACUC-RC2015-23).

*T. parva* schizonts proliferate in the white blood cells, whereas piroplasms develop in the red blood cells of the host. Schizont-infected bovine lymphocytes are easily cultured *in vitro*. Thus, the schizont parasites were purified from approximately 2 × 10^8^ cells that were obtained from the *in vitro* established *T. parva* (Muguga) schizont-infected bovine peripheral blood mononuclear cell line TpM 3087 at the International Livestock Research Institute, as previously described (16, 17). Four different schizont purification assays were performed. The piroplasm parasites were purified from *T. parva* (Muguga)–infected calf blood when the parasitemia reached 3–70%, as previously described (18). Three piroplasm purification experiments were conducted. For this study, piroplasms and schizonts were *T. parva* Muguga stabilate 3087, previously described by Tonui et al. (16).

### RNA Extraction and cDNA Library Preparation

Purified schizont and piroplasm parasite samples were processed for total RNA extraction and purification using the RNAzol^®^ RT isolation kit following the manufacturer's instructions (Sigma-Aldrich, USA). *T. parva* total RNA contains an abundant ribosomal RNA that migrates as a strong band between the 18 and 28S host bovine rRNA on a 1.5% agarose gel electrophoresis (19). We used this approach to verify that RNA samples contained *T. parva* RNA and were not degraded before we proceeded with subsequent analyses. Then, isolated RNA was quality-checked and quantified using the Nanodrop^®^-1000 spectrophotometer (Nanodrop Technologies, Delaware, USA). We further checked the integrity of the RNA using 1.2% agarose RNA gel, as described previously (20). The poly(A)^+^RNA was purified from the total RNA (16). The integrity and quantity of poly(A)^+^RNA were checked as above; then, 10 ηg of RNA was used for each library. Normalization of the schizont and piroplasm poly(A)^+^RNA was done using Ambion^®^ ERCC Spike-In Control, as described before (15). The TruSeq stranded total RNA Kit (Illumina Inc., USA) was used for the library preparation according to manufacturer instructions. The library concentration was checked using the Qubit^®^ (Thermo Scientific, USA) broad range and high sensitivity reagents, while the integrity was checked using the Agilent Bioanalyzer 2200 TapeStation system. For the sequencing, each library was diluted before being pooled for sequencing to avoid over-clustering errors on the sequencer.

### Sequencing and Differential Expression Analysis

Paired-end RNA sequencing was done at the Biosciences eastern and central Africa-International Livestock Research Institute (BecA-ILRI) Hub using an Illumina MiSeq sequencer following manufacturer guidelines. The reads obtained were transferred to the ILRI High-Performance Computing (HPC) server for bioinformatics data analysis. The nucleotide sequence data reported in this study are available in the NCBI database under the accession number PRJNA604662. The quality control of the raw reads was done using FastQC 0.11.5 (21). The reads were cleaned, and the adapters were trimmed using trimmomatic/0.38 and cutadap 1.16 (22, 23). An index of the *T. parva* transcriptome was then built, based on the original genome annotation (11). The trimmed reads were used for the mapping against the built transcriptome using Kallisto version 0.43.0 (24). The *T. parva* reference transcriptome (accession no. GCF_000165365.1_ASM16536v1) was retrieved from NCBI GenBank. An RNA-seq pipeline was developed using a custom Python script 3.7 for the analysis from the quality control to the quantification. The read counts were normalized to transcripts per kilobase million (TPM). The count table (h5 format) was exported to R for the gene expression analysis. In order to confirm the statistically significant changes in gene expression and the complete data set for different stage pairwise comparisons, gene expression analysis was conducted. Bioconductor DEseq2 (25) based on the negative binomial distribution packages was used to identify differentially expressed genes at the different parasite stages. Genes were regarded to be differentially expressed when the q value cutoff (FDR adjusted *p*-value using Benjamini–Hochberg model) was lower than 0.05. The differentially expressed genes were plotted using the Bioconductor EnhancedVolcano package in R (26).

The quality control was also performed on the unmapped reads. They were *de novo* assembled to form contigs using Trinity v2.6.6 (27). The transcripts were then blasted against the non-redundant (nr) GenBank database. The hit contigs from Trinity were mapped back to the *T. parva* reference genome.

### Functional Enrichment of the Differentially Expressed Genes and Vaccine Candidate Antigen Prediction

Functional annotation of the significant differentially expressed genes between the infection stages was performed using the Database for Annotation, Visualization and Integrated Discovery (DAVID Bioinformatics Resources 6.8, NIAID/NIH). Gene Ontology (GO) term enrichment was analyzed for functional classification of selected up- and downregulated genes in each of the two parasite stages. *In silico* search of the N-terminal signal peptide (SP), trans-membrane domain (TMD), nuclear localization signal (NLS), C-terminal glycosylphosphatidylinositol (GPI) anchor signal, prediction of protein function, and non-classical protein secretion were analyzed using bioinformatics tools SignalP 4.0 (28), Protter server (29), PredictProtein (30), PredGP (31), and SecretomeP 2.0 server (32), respectively, as described previously (16, 33).

Genes having similar expression patterns to known *T. parva* vaccine antigens were also identified using PAM unsupervised clustering algorithm in R with k = 50 (34, 35). Antibody epitope residue scores (B-cell epitopes) were predicted using BepiPred Linear Epitope Prediction 2.0 (http://tools.iedb.org/bcell) for genes encoding proteins predicted to be localized on the surface of the parasite (having a predicted TMD or GPI anchor). The prediction tools are available on the Immune Epitope Database Analysis Resource (IEDB) (http://tools.iedb.org/). The Kyoto Encyclopedia of Genes and Genomes (KEGG) database (https://www.genome.jp/kegg/) (36) was used to predict the metabolic pathway of the proteins, whereas MDLocProtein (37), YLoc (38), and WOLF PSORT II (39) were used to identify the subcellular localization of the proteins having a predicted domain.

### Preliminarily Validation of Candidate Vaccine Antigens Using an Enzyme-Linked Immunosorbent Assay

Selected sera from naturally infected and ECF-positive cattle, identified using a polymorphic immunodominant molecule (PIM)–based indirect enzyme-linked immunosorbent assay (ELISA) test (40, 41), were used to assess the antigenicity of potential vaccine antigens and predicted epitopes. The antigen open reading frame was PCR amplified, cloned into a pET-32a+ plasmid vector, then over-expressed in *Escherichia coli* BL21, and affinity-purified as previously described (42). The BepiPred-predicted peptide epitopes were synthesized and lyophilized (Pepscan, Netherlands). Peptides were dissolved in 1 ml of 50% (v/v) analytical grade acetonitrile/water (Applied Biosystems) to a final concentration of 2 mg/ml (2 μg/μl). The ELISA was performed as previously described (41) with a serum dilution of 1:5. Each serum was tested three times. The plates were read at 405 nm with the Immunoskan ELISA reader using the program EDI. Data analysis was performed using the ELISA program integrated into the ELISA reader and the results were presented as percent positivity (PP). Predetermined bovine positive (*PP* ≥ 80) and negative control (*PP* ≤ 10) sera and antigen (lysate from culture of *E. coli* BL21 containing pET-32a+ plasmid vector expressing thioredoxin fusion tag) were included in each ELISA test plate. Optical density (OD) readings from the reference positive control sera were used to compute the PP for the test sera. *PP* values of 20 and above were considered positive for *T. parva* as previously described (41, 43). Selected ECF-positive sera were collected in 2017 at Gitega and Gankuzo, Burundi, and stored at BecA-ILRI Hub (Courtesy of Dr Lionel Nyabongo, ISABU, Burundi).

## Results

### Kallisto Read Mapping to the Reference Transcriptome

Previously, we showed that Kallisto generates more mapped reads than TopHat2 (16). Therefore, Kallisto was used to map schizont and piroplasm paired-end reads to the *T. parva* reference transcriptome and to quantify transcript abundance in each sample replicate (Supplementary Data File 1). Out of 3,717,927 schizont trimmed reads, the average percentage of mapped reads for the four technical replicates was 60%, while out of 3,143,345 piroplasm trimmed reads, the average percentage of mapped reads for the three technical replicates was 85% (Table 1). The schizont reads mapped to transcripts of 3,891 genes, whereas reads from the piroplasm mapped to transcripts of 3,887 genes. In total, 4,061 different protein coding genes were identified by the combined schizont and piroplasm reads out of a possible 4,084 protein coding genes predicted by the recent re-annotation of the *T. parva* genome (44).

**Table 1:** Percentage of reads mapped to the reference genome transcriptome using Kallisto and number of mapped genes.

	**Life cycle stage**	
	**Schizont**	**Piroplasm**
Number of sample replicates	4	3
Total trimmed reads	3,717,927	3,143,345
Mapped reads	60%	85%
Mapped genes	3,891	3,887
Combined total genes mapped	4,061	
*Theileria parva* protein coding genes^a^	4,084	

*Using p67 (TP03_0287) as benchmark, a gene with a transcripts per kilobase million (TPM) value ≤ 2.2 was considered not expressed in the stage studied*.

a *4,084 proteins are predicted to be encoded by the re-annotated T. parva genome (44)*.

**Figure 1:**
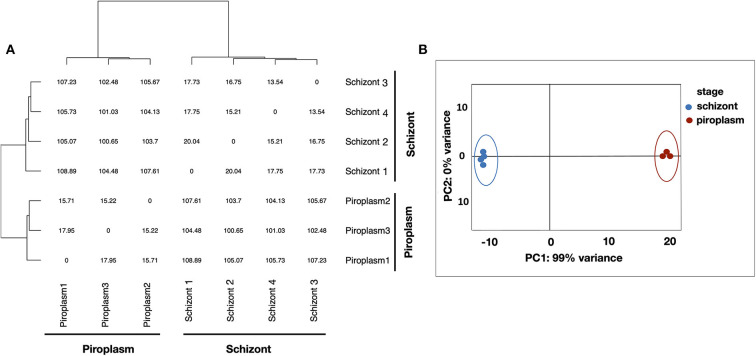
Clustering analysis of samples. **(A)** Samples' distance matrix of replicates clustering schizont and piroplasm replicates according to the life cycle stages. **(B)** Principal component analysis (PCA) of the replicates showing a variance of 99% between the schizont (blue) and piroplasm replicates (red).

Kallisto was used to normalize the counts in TPM, to avoid biases induced by external factors. The normalization is essential to ensure that the expression distributions of each sample are similar across the entire experiment to account for differences in gene length and in sequencing depth across replicates. The gene expression data were displayed using clustering methods that group genes and sample replicates together based on the expression pattern similarities. Samples' distance matrix of replicates (Figure 1A) and principal component analysis (PCA) analyses (Figure 1B) clustered the replicates according to the life cycle stages and the divergence between each pair of samples (Figure 1). No significant difference was observed among replicates of the same life cycle stage. The four schizont replicates clustered together, while the three piroplasm replicates also clustered together.

### Gene Expression in the Schizont and Piroplasm Stages of *T. parva*

**Figure 2:**
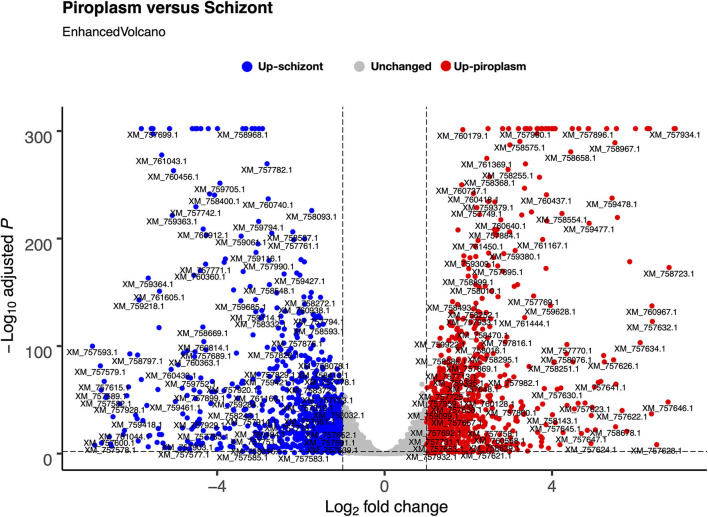
Differential gene expression between schizont and piroplasm stages using Bioconductor EnhancedVolcano package in R. The graph shows the distribution of the upregulated genes between schizont (blue dots) and piroplasm (red dots) stages with the exception of the non-differentially expressed or unchanged genes (gray dots). Each dot in the EnhancedVolcano diagram represents one differentially expressed gene. The x-axis represents the Log2 fold change, and the y-axis shows the –Log10 adjusted *P*-value.

We used the sporozoite antigen p67 (TP03_0287), which is not expressed in the schizont stage, as a benchmark to set the minimum TPM expression threshold above 2.2 (TPM > 2.2). Therefore, a gene with a TPM value ≤ 2.2 was considered not expressed in the life cycle stages studied. Pairwise comparison was performed in the piroplasm relative to the schizont stage. Thus, 3,279 genes were differentially expressed between the two stages, with roughly half of them being significantly upregulated in each stage (Figure 2). We found that 1,624 (51%) genes were upregulated, whereas 1,656 (49%) were downregulated in the piroplasm with reference to the schizont (Figure 2).

**Figure 3:**
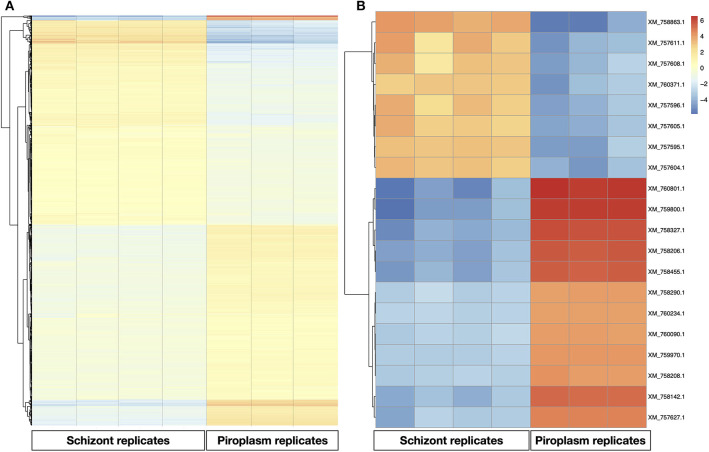
Gene expression profile in the schizont and piroplasm life cycle stages. **(A)** Heatmap of all the differentially expressed genes. **(B)** Heatmap of the top 20 most variable genes between schizont replicates and piroplasm replicates. The genes were clustered into two groups: from genes XM_760801.1 to XM_757627.1 where genes were upregulated in piroplasm and downregulated in schizont, and from XM_757604.1 to XM_758863.1 where genes where upregulated in schizont and downregulated in piroplasm. The genes were clustered by hierarchical clustering within each horizontal partition and replicates for the infection stages within each vertical partition by similarity. Color red denotes high expression, yellow denotes stable expression, and blue denotes low expression. The colors are scaled per row.

A heatmap of the differentially expressed genes was also generated with clustering methods that group genes and replicates by gene expression profile, showing once again that replicates from the same life cycle stage are most similar to each other (Figure 3A). A heatmap was also performed to identify the profile of the top 20 most variable genes across the two stages studied, which showed that 8 were upregulated in the schizont stage (Figure 3). These were: XM_757611.1 (TP05_0035), XM_757608.1 (TP05_0032), XM_760371.1 (TP02_0896), XM_757595.1 (TP05_0019), XM_757604.1 (TP05_0028), XM_757605.1 (TP05_0029), XM_757596.1 (TP05_0020), and XM_758863.1 (TP04_0321). Some of them code for hypothetical proteins with SP and/or TMDs such as TP05_0020, TP05_0035, and TP05_0032. The remaining 12 most differentially expressed genes were downregulated in the schizont and highly expressed in piroplasm [XM_760801.1 (TP01_0367), XM_759800.1 (TP02_0327), XM_758327.1 (TP03_0400), XM_758142.1 (TP03_0217), XM_758455.1 (TP03_0520), XM_758206.1 (TP03_0281), XM_760234.1 (TP02_0760), XM_757627.1 (TP03_0905), XM_759970.1 (TP02_0497), XM_758208.1 (TP03_0283), XM_760090.1 (TP02_0617), and XM_758290.1 (TP03_0363)]. Genes TP03_0281 and TP03_0283 encode cysteine proteases containing a TMD. In contrast, genes TP01_0367, TP02_0327, TP02_0617, TP03_0008, TP03_0217, TP03_0363, TP03_0400, TP03_0520, and TP03_0905 all code for hypothetical proteins with a predicted SP, except for TP03_0905, which contains one TMD.

Stage-specific genes were also identified and are presented in Supplementary Data File 2. The top 20 most highly expressed stage-specific genes are presented in Table 2. These genes will be further investigated by qRT-PCR.

**Table 2:** Top 20 highest expressed stage-specific genes.

**GenBank acc. no**.	**Gene ID**	**TPM values**		**Domains**	**Product name**
		**Schizont**	**Piroplasm**		
XM_758142.1	TP03_0217	0	3,318	SP	Hypothetical protein
XM_757627.1	TP03_0905	0	924	3 TMD	Hypothetical protein
XM_758244.1	TP03_0319	0	463	(-)	Hypothetical protein
XM_757628.1	TP03_0906	0	156	5 TMD	Hypothetical protein
XM_760967.1	TP01_0540	0	130	(-)	Hypothetical protein
XM_757613.1	TP05_0037	2,060	0	2 TMD	Hypothetical protein
XM_757596.1	TP05_0020	1,714	0	3 TMD	Hypothetical protein
XM_757611.1	TP05_0035	1,496	0	TMD	Hypothetical protein
XM_757616.1	TP05_0040	1,271	0	2 TMD	Hypothetical protein
XM_757610.1	TP05_0034	992	0	2 TMD	Hypothetical protein
XM_757608.1	TP05_0032	977	0	1 TMD	Hypothetical protein
XM_757584.1	TP05_0008	689	0	(-)	Ribosomal protein L14, putative
XM_757605.1	TP05_0029	586	0	(-)	DNA-directed RNA polymerase beta' chain
XM_757598.1	TP05_0022	567	0	1 TMD	Hypothetical protein
XM_757618.1	TP05_0042	433	0	(-)	DNA-directed RNA polymerase subunit beta
XM_757599.1	TP05_0023	420	0	(-)	ClpC molecular chaperone, putative
XM_757604.1	TP05_0028	418	0	(-)	DNA-directed RNA polymerase subunit beta (PEP)
XM_757591.1	TP05_0015	409	0	(-)	50S ribosomal protein L36, apicoplast
XM_760366.1	TP02_0891	320	0	(-)	Hypothetical protein
XM_757617.1	TP05_0041	277	0	1 TMD	Hypothetical protein

*Expression threshold = TPM >2.2. (Genes with a TPM ≥ 2.2 were considered as not expressed, and their TPM values were set to 0). SP, signal peptide; TMD, trans-membrane domain; (-), none*.

### The Top 5 of the 20 Most Highly Expressed Genes Occurred in the Schizont Stage

The level of expression of the 20 most highly expressed genes identified in this study varied from 3,594 TPM for TP03_0050 (in the piroplasm) to 69,380 TPM for TP04_0321 (in the schizont) (Table 3). Only six of them encode for proteins with specific domains, including four (TP01_0367, TP02_0327, TP03_0400, and TP01_1056) containing an N-terminal SP. The five most highly expressed genes occurred in the schizont stage, namely TP04_0321, TP04_0322, TP04_0404, TP04_0675, and TP04_0677, all of which encode histone proteins. However, most of the 20 most highly expressed genes were preferentially expressed in the piroplasm stage rather than in the schizont. TP04_0675 and TP04_0677 had the same TPM counts (Table 3). A BLAST search revealed that the two genes code for the same protein, a 103-amino-acid-long putative histone H4.

**Table 3:** Top 20 highest expressed genes.

**GenBank acc**.	**Gene_ID**	**TPM values**		**Name**	**Domains**
		**Schizont**	**Piroplasm**		
XM_758863.1	TP04_0321	69,380	145	Histone H3	(-)
XM_758864.1	TP04_0322	55,504	1,307	Histone H2A	(-)
XM_758946.1	TP04_0404	51,621	1,830	Histone H2B-III	(-)
XM_759218.1	TP04_0675	48,611	831	Histone H4	(-)
XM_759220.1	TP04_0677	48,611	831	Histone H4	(-)
XM_760801.1	TP01_0367	13	39,381	Hypothetical protein	SP, GPI
XM_758723.1	TP04_0181	255	29,488	Hypothetical protein	(-)
XM_758206.1	TP03_0281	23	22,581	Cysteine proteinase	1 TMD
XM_759800.1	TP02_0327	9	22,413	Hypothetical protein	SP
XM_759624.1	TP02_0148	2,748	15,381	Heat shock protein 70	(-)
XM_760882.1	TP01_1228	15,366	13,213	Hypothetical protein	(-)
XM_758327.1	TP03_0400	4	8,567	Hypothetical protein	SP, GPI
XM_758444.1	TP03_0509	1,933	7,550	Hypothetical protein	(-)
XM_761484.1	TP01_1056	413	6,930	32 kDa surface antigen	SP, 1 TMD, GPI
XM_759970.1	TP02_0497	42	6,586	AP2/ERF domain	(-)
XM_760881.1	TP01_1233	6,395	704	Hypothetical protein	(-)
XM_758455.1	TP03_0520	4	5,268	Hypothetical protein	(-)
XM_758208.1	TP03_0283	25	3,898	Cysteine proteinase	1 TMD
XM_759756.1	TP02_0283	1,010	3,792	60S ribosomal protein L39	(-)
XM_757976.1	TP03_0050	568	3,594	60S ribosomal protein L24	(-)

*GPI, glycosylphosphatidylinositol anchor; (-), none*.

### Functional Annotation of the Upregulated Genes in the Piroplasm Stage

**Figure 4:**
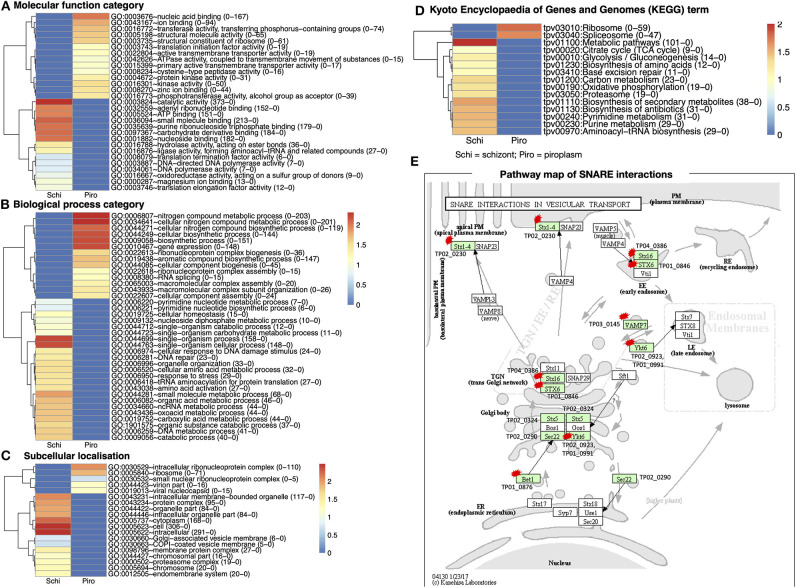
Gene Ontology (GO) functional classification for differentially expressed genes in the two developmental stages studied. **(A)** Molecular function category. **(B)** Biological process category. **(C)** Subcellular localization. **(D)** Kyoto Encyclopedia of Genes and Genomes (KEGG) database of the categorized genes downregulated in schizont and upregulated piroplasm. Genes without ontology information are not included. The proportion number of each GO and KEGG term of schizont and piroplasm is, respectively, presented in the parentheses in front of the GO and KEGG terms. Red color indicates most enriched (2), and blue, no enrichment (0). The expression level is color coded: red for over-expressed, white for unchanged. **(E)** Pathway map of SNARE interactions in vesicular transport. This pathway is annotated with most of the hypothetical proteins among all the referred pathways. The arrows in the pathway indicate the molecular interaction or reaction. The green boxes are hyperlinked to identified genes (encoded proteins) involved in the reaction, and the green box with a red star is hyperlinked to the hypothetical proteins involved in the pathway. Schi represents schizont stage, and Piro, piroplasm.

GO of differentially expressed genes for the two stages in the bovine white and red blood cells was classified according to three major categories on DAVID: molecular functions, biological processes, and cellular component. There were 2,116 functional categories assigned to over-expressed genes in the piroplasm relative to the schizont. The GO categories associated with upregulated genes in the piroplasm stage referred to 711 molecular functions, 1,194 biological processes, and 211 subcellular localizations (Figures 4A–C).

There were 711 molecular functional categories assigned to genes upregulated in the piroplasm relative to the schizont. The molecular function categories that were most broadly enriched included nucleic acid and ion binding, cysteine peptidase, protein kinase, and translation initiation factor activity (Figure 4A), which included ATP binding, catalytic activity, purine ribonucleoside triphosphate binding, and carbohydrate derivative binding.

Biological process categories assigned to genes upregulated in the piroplasm (1,194) included genes involved in the nitrogen metabolic process, cellular biosynthesis process, and gene expression. In contrast, the biological processes such as single organism process, response to stress, and amino acid activation were significantly enriched in the schizont (Figure 4B). Cellular component categories assigned to genes upregulated in the piroplasm (211) were most broadly enriched in the intracellular ribonucleoprotein complex and ribosome (Figure 4C).

To better understand the potential function of the differentially expressed genes, pathway analysis was performed. A difference was observed between up- and downregulated genes, in that 106 upregulated genes and over thrice as many (366) downregulated genes were associated to pathways in the KEGG database. The enriched pathways are shown in Figure 4D. The top signaling pathways annotated, to which genes upregulated in the piroplasm stage belong, included ribosome and spliceosome. To contribute to the knowledge of parasite genes potentially involved in vesicular transport signal pathways, we identified in the differentially expressed genes SNARE (soluble N-ethylmaleimide–sensitive factor attachment protein receptor) protein homologs using KEGG Mapper (Figure 4E); targets identified are primarily genes encoding hypothetical proteins and mostly downregulated in the schizont. The list of genes associated to each GO term and KEGG term is presented in Supplementary Data File 3.

### Genes With Functional Domains and Expression Patterns Similar to Known *T. parva* Antigen Genes

To identify genes that have a similar expression pattern to known vaccine antigen-coding genes, gene expression profiles and functional domains and motifs of these known antigens were first generated (Table 4). The analysis was expanded to include data previously reported for two tick vector stages (sporoblast and sporozoite) as well as the schizont (16). These known antigens are differentially expressed across the infection stages. However, except for the p67, which was not expressed in the piroplasm stage (0 TPM), all the other known antigens were differentially expressed across all the stages studied.

**Table 4:** Expression profile of known *T. parva* antigens.

**Locus tag ID**	**Antigen name**	**TPM values**					**Product name**	**Domains**
		Sporoblast^*^	Sporozoite^*^	Schizont^*^	**Schizont**	**Piroplasm**		
TP03_0849	Tp1	111	990	155	320	201	CD8+ T-cell target antigen	SP
TP01_0056	Tp2	309	1,427	625	533	39	CD8+ T-cell target antigen	SP
TP01_0868	Tp3	280	61	145	25	176	CD8+ T-cell target antigen	SP
TP03_0210	Tp4	369	431	242	309	199	T-complex protein 1 subunit eta	(-)
TP02_0767	Tp5	702	503	292	249	175	Translation initiation factor eIF-1A	(-)
TP01_0188	Tp6	475	261	275	248	151	Prohibitin	SP
TP02_0244	Tp7	2,363	2,324	803	2,034	1,067	Heat shock protein 90	(-)
TP02_0140	Tp8	3,146	1,658	4,315	3,044	2,740	Cysteine proteinase	SP
TP02_0895	Tp9	273	6,384	1,497	708	5	CD8+ T-cell target antigen	SP
TP04_0772	Tp10	954	515	1,090	300	290	Coronin	(-)
TP03_0287	p67	17,464	840	21	3	0	Hypothetical protein	SP, 1 TMD
TP04_0051	PIM	322	3,361	961	2,661	905	Polymorphic immunodominant molecule	SP, 3 TMD
TP01_0939	gp34	24	261	113	101	113	Hypothetical protein	SP, 1 TMD
TP04_0437	p104	15	11,151	539	395	39	104 kDa microneme/rhoptry antigen	SP, GPI
TP03_0445	PCNA	1,225	102	271	337	102	Proliferating cell nuclear antigen	(-)
TP02_0600	PCNA	710	49	420	314	181	Proliferating cell nuclear antigen	NLS

* *Data from Tonui et al. (16)*.

*The p67 TPM value was considered as background and was subtracted from the expression value of each antigen. GPI, GPI anchor; NLS, nuclear localization signal; PIM, polymorphic immunodominant molecule; (-), none*.

**Figure 5:**
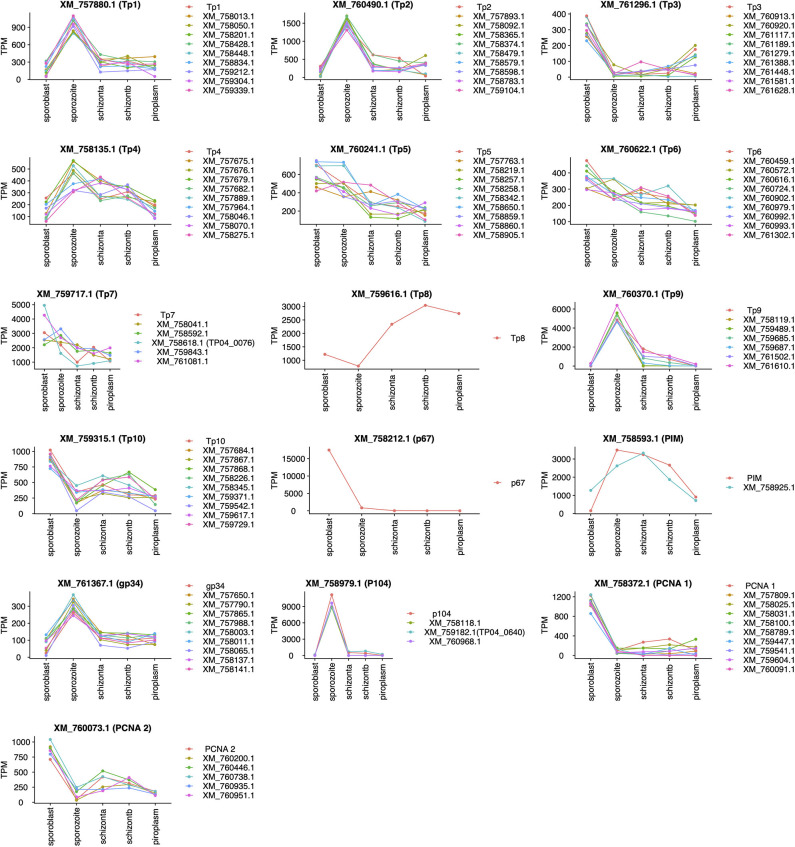
Genes with expression profiles similar to known *Theileria parva* antigens' genes. Sporoblast, sporozoite, and schizont_a are transcriptome data collected from our previous work (16), whereas schizont_b and piroplasm are transcriptome data generated from this study. Each line in a unique color represents a specific gene with its GenBank accession number. Each plot represents the profiling of genes (GenBank accession number listed) having a similar expression profile to the known antigen (top of the plot).

Unsupervised clustering using the PAM clustering algorithm in R was performed to identify genes that cluster with, and thus have a similar expression pattern to, known antigens (Figure 5; Supplementary Data File 4 Sheet 1). Several genes had a similar expression pattern to known antigens, including 10 or more that were similar to Tp1, Tp2 and Tp3, Tp4, Tp5, Tp6, Tp10, gp34, and PCNA 1 (TP02_0600); 6 that were similar to Tp9; 5 similar to both PCNA 2 (TP03_0445) and Tp7; 3 similar to p104; and 1 gene similar to PIM. However, we did not find any gene with similar expression patterns to Tp8 and p67. Those with an SP, TMD, and/or GPI anchor are shown in Supplementary Data File 4 Sheet 2.

Vaccination against ECF using the major *T. parva* sporozoite surface protein p67 can induce antibody-based immune protection in up to 50% of vaccinated animals (3, 45). To support the identification of additional candidate vaccine antigens able to induce protective antibodies like p67, *T. parva* genes encoding proteins containing TMDs or GPI anchors were analyzed *in silico* using the epitope prediction algorithm BepiPred Linear Epitope Prediction 2.0 for the presence of epitopes that are targets of antibodies (Supplementary Data File 5). Using p67 antigen as the benchmark, the top 20 proteins among the candidate vaccine proteins predicted to be localized in the parasite plasma membrane (Supplementary Data File 5) were ranked for their antigenic propensity and/or probability to contain antibody epitopes based on the score of the predicted peptide epitopes, which varied between 0.749 (XP_763541.1) and 0.566 (XP_764275.1), compared to the p67 antigen score of 0.658 (Table 5) or PIM antigen of 0.650.

**Table 5:** Top 20 *T. parva* surface proteins with high antibody epitope prediction values and with expression similar to known antigen.

**Gene ID**	**Protein ID**	**SP**	**SZ**	**Sca**	**Scb**	**PRM**	**ESA**	**DM**	**Name**	**SLP**	**NE**	**HPRS**
TP03_0513	XP_763541.1	150	800	335	296	199	Tp1	5 TMD	HP	PM	7	0.749
TP02_0615	XP_765181.1	345	174	14	9	31	Tp3	7 TMD	RING-type domain-containing protein	PM	20	0.738
TP03_0544	XP_763572.1	32	1,568	360	203	98	Tp2	10 TMD	HP	PM	6	0.717
TP01_1200	XP_766721.1	295	22	97	49	13	Tp3	7 TMD	Phosphodiesterase	PM	11	0.711
TP02_0232	XP_764798.1	503	1,387	266	274	19	Tp2	11 TMD	HP	PM	10	0.709
TP01_0509	XP_766029.1	104	339	134	163	99	gp34	SP, 1 TMD	HP	ES	13	0.709
TP03_0168	XP_763186.1	91	437	639	358	108	Tp4	SP, 1 TMD	HP	PM	88	0.703
TP03_0165	XP_763183.1	19	366	123	131	3	gp34	9 TMD	ABC transporter	NS	34	0.702
TP01_1013	XP_766534.1	146	310	96	88	165	gp34	SP, 7 TMD	MtN3/RAG1IP protein	PM	9	0.700
TP04_0448	XP_764083.1	276	22	28	25	76	Tp3	8 TMD	HP	PM	21	0.699
TP01_0506	XP_766026.1	145	556	242	195	162	Tp4	SP, 1 TMD	GOLD domain-containing protein	ES	7	0.679
TP04_0076	XP_763711.1	4,952	608	727	907	1,097	Tp7	2 TMD	HP	PM	2	0.677
TP02_0183	XP_764747.1	62	288	151	112	97	gp34	9 TMD	HP	PM	21	0.674
TP04_0803	XP_764440.1	98	370	318	236	205	Tp4	8 TMD	TPT domain-containing protein	PR	11	0.659
TP03_0287	XP_763305.1	17,465	840	21	3	2	p67	SP, 1 TMD	HP	PM-ES	18	0.658
TP03_0175	XP_763193.1	1,063	42	21	90	147	PCNA	SP, TMD	HP	ES	18	0.658
TP04_0907	XP_764544.1	3	336	102	91	6	gp34	18 TMD	HP	PR	23	0.652
TP03_0895	XP_763368.1	61	308	432	256	121	Tp4	12 TMD	Folate/biopterin transporter	PM	17	0.650
TP03_0455	XP_763475.1	238	17	61	64	119	Tp3	SP, 6 TMD	HP	PM	10	0.645
TP04_0640	XP_764275.1	136	8,957	671	794	202	p104	1 TMD	HP	PM	2	0.566

^*^p67 antigen is used as reference for the prediction value. SP, sporoblast; SZ, sporozoite; SCa, schizont a; Scb, schizont b; PRM, piroplasm; DM, domain; ES, extracellular localization; ESA, expression similar to known antigen; HP, hypothetical protein; HPRS, highest epitope predicted residue scores; NE, number of predicted epitopes; PM, plasma membrane; NS, nucleus; PR, peroxisome; SLP, subcellular localization prediction.

### Unmapped Reads Mainly Originate From the Bovine Genome

**Figure 6:**
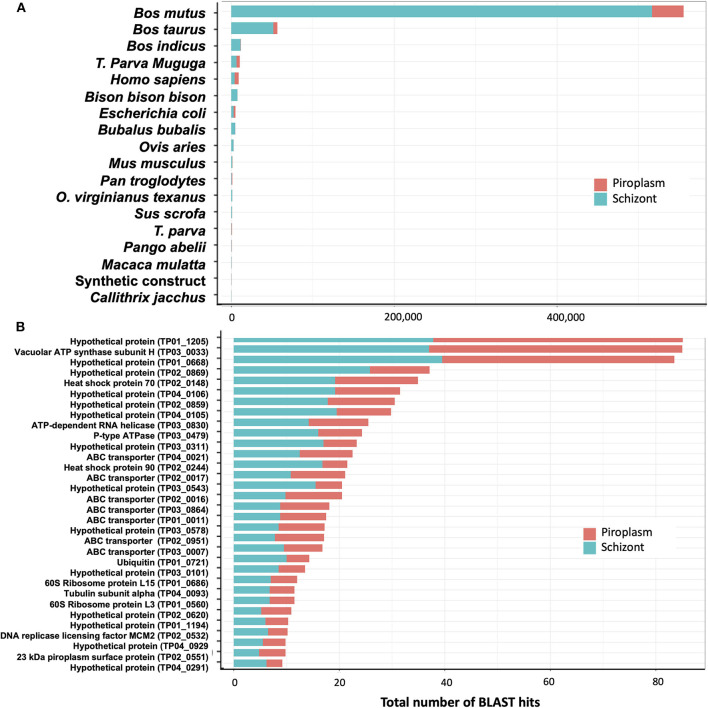
Analysis of unmapped reads. **(A)** Count of BLAST hits to unmapped reads. **(B)** Counts of BLAST hits of unmapped reads to *T. parva* genome.

*De novo* assembly of unmapped reads from schizont samples, i.e., 40% of total trimmed reads (Table 1), using Trinity, generated 10,823 contigs. BLAST searches for sequence similarity were performed using the new contigs. Most of the hits mapped to mammalian genomes in the family Bovidae, including *Bos mutus, Bos taurus, Bos indicus*, and *Bison bison* (Figure 6A). By assuming that all these genes are more likely orthologs to the bovine host genes, then in total, 71% of the schizont stage unmapped reads hit the bovine genome. The second large hit was the *T. parva* genome to which 23% of the total blast hits mapped. Other blast hits included bacteria (*E. coli*) and synthetic constructs, which may be plasmids from the *E. coli* above. Trinity *de novo* assembly of piroplasm unmapped reads produced 2,064 contigs, of which 90% mapped to bovine and about 5% to *Theileria* genomes. The contigs mapped to the *T. parva* genome identified 32 genes (Figure 6B). It is likely that the structure of those genes was not correctly identified in the original annotation and thus was incomplete or had gaps in the reference transcriptome used for the mapping, as we demonstrated previously (16). These have now been re-annotated accordingly (44). Most of the 32 genes identified by both schizont and piroplasm contigs of unmapped reads were hypothetical proteins. Known genes coded for ABC transporters, ribosomal proteins, heat shock proteins (including heat shock protein 90, known as Tp7 or TP02_0244), or 23 kDa piroplasm surface protein TP02_0551, among others (Figure 6B).

### ELISA Validation of Potential Vaccine Antigens

**Figure 7:**
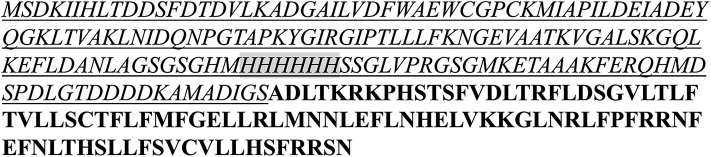
Amino acid sequences of the recombinant fusion protein TP04_0076F. The fusion protein contains 260 aa (single letter peptide) with the vector portion underlined and the 6x His tag used for affinity purification shadowed. The TP04_0076 portion is bolded, and the methionine (M) start codon was not included in the fusion protein.

We chose two candidate vaccine antigens among the predicted parasite plasma membrane proteins (Table 5) to evaluate their immunogenicity potential. TP04_0076 and TP04_0640 genes were selected because they may encode plasma membrane proteins, have strong predicted antibody epitope residue scores [0.677 and 0.566, respectively (Table 5)], and encode very small proteins (only 96 and 114 amino acids, respectively) that may therefore be easy to express in *E. coli* and purify. Furthermore, TP04_0076 (XM_758618.1) has an expression profile similar to Tp7, and that of TP04_0640 (XM_759182.1) is similar to the p104 antigen gene (Figure 5). TP04_0640 was proposed as a potential target for the development of anti-*Theileria* drugs (16). Recombinant protein was generated only for TP04_0076. The TP04_0076 ORF was cloned into pET-32a+, over-expressed as a recombinant fusion protein in the *E. coli* system, and affinity-purified. The fusion protein was termed TP04_0076F (Figure 7). We procured synthetic peptides corresponding to the following predicted antibody-targeted epitopes: TP04_0076ep1 (19-mer: MADLTKRKPHSTSFVDLTR) in TP04_0076, and TP04_0640ep1 (19-mer: PDRFFNKIGIIYYPSKHWS) and TP04_0640ep2 (30-mer: ERTKHPRLDSFDSMIDEYSTVENDGGIMYF), both in TP04_0640. As previously described (16), TpMuguga_04g00640 encodes a protein that is 50 amino acids longer than that of TP04_0640, the corresponding locus predicted in the original 2005 genome annotation, as it now encodes a protein of 114 amino acid residues.

**Figure 8:**
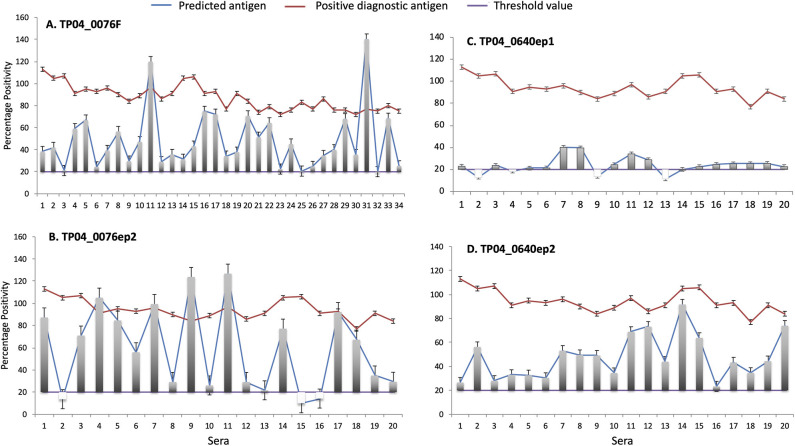
Enzyme-linked immunosorbent assay (ELISA) evaluation of the antigenicity of **(A)** TP04_0076 gene product (recombinant protein TP04_0076F) and predicted antibody target epitopes of TP04_0640 and TP04_0076 gene products (synthetic peptides **(B)** TP04_0076ep2, **(C)** TP04_0640ep1, and **(D)** TP04_0640ep2). A panel of 34 predetermined bovine positive controls with strong percent positivity (*PP* ≥ 80) to PIM antigen (red line) were included in the analysis. The PP results of each of the four recombinant proteins and synthetic peptides tested (blue line) are shown in separated panels. The green line represents the cutoff, and an assay with a *PP* ≥ 20 (*PP* ≥ 20) is considered positive.

The antigenicity of both the purified recombinant fusion protein and synthetic peptides was tested using cattle sera that exhibited a strong positive response in a PIM-based ELISA. In total, and since we lacked sufficient volume to test all 34 strong positive sera against all epitopes, TP04_0076F was tested with 34 sera, and TP04_0076ep2, TP04_0640ep1, and TP04_0640ep2, with 20 sera (Figure 8). Three negative control sera were included (Supplementary Data File 6).

All the 34 infection sera analyzed were positive with the recombinant fusion protein TP04_0076F, with PP ranging from 20 to 140 PP (cutoff value: *PP* ≥ 20). Equally, all the sera tested with the synthetic peptide epitope TP04_0640ep2 produced a positive antibody reaction. Six sera showed higher levels of antibody reaction to the recombinant protein TP04_0076F and derived epitope TP04_0076ep1 than the positive control PIM which is being used as a diagnostic antigen (Figures 8A,B). In contrast, all the sera tested with the TP04_0640ep2 epitope gave positive antibody reactions but at lower levels than PIM (Figure 8D). Out of 20 sera tested with TP04_0640ep1, 16 (80%) were positive (Figure 8C). The OD reading of each antigen and the positive and negative control sera and antigen are presented in Supplementary Data File 6. All the antigens tested produced a very weak or no reaction to the negative control sera. The presence of antibodies in the sera was a preliminary indication of the antigenicity of these potential antigens.

## Discussion

This research was undertaken in order to enhance our knowledge and understanding of gene expression across *T. parva* life cycle stages, specifically from schizont to piroplasm forms, and in this way improve predictive analysis of their structure, location, role, and function that may lead to the identification of novel therapeutic targets.

A comparison of the transcriptome data from next-generation sequencing of the two stages pointed to 3,279 differentially expressed genes between the schizont and the piroplasm stage, out of 4,061 protein coding genes with our expression data, with roughly half of them being significantly upregulated in each of these two stages that take place, respectively, in the host white blood cells and red blood cells. To capture the whole expression profile of the parasite, we summarize in Table 6 the transcriptome data sets of the different life cycle stages, by combining our current data with a similar study previously conducted on the sporoblast, sporozoite, and schizont stages (16). Shaw et al. (42) have proposed that *T. parva* uses repeatedly a single set of genes (termed “cassettes”) throughout its life cycle with the replacement of only a few each time to allow for small differences in reproduction (46). These genes are regulated by a limited number of promoter motifs (47). Consistent with this scenario, the results presented here and in our previous work show that almost all genes are expressed in most stages, albeit across a wide range of expression levels, with only about 200 being stage-specific. It worth noting that, as the parasite developed from sporoblast to sporozoite and then schizont stages, the number of differentially expressed genes increased (16) and then slightly decreased from the schizont to the piroplasm stages (this study).

**Table 6:** Number of *T. parva* genes differentially expressed in the tick vector and bovine host.

	**Life cycle stages**			
**Gene status**	**Sporoblast^a^**	**Sporozoite^a^**	**Schizont^a^**	**Piroplasm^b^ (compared to schizont^b^)**
Downregulated	1,136	1,517	4	1,623
Upregulated	1,332	1,626	3,862	1,656
Total differentially expressed	2,468	3,143	3,866	3,279
Total genes mapped	3,924			4,061

a *Data from Tonui et al., (16)*.

b *This study*.

The top five most highly expressed genes are over-expressed in the schizont stage and encode histone protein family members (Table 3). However, Tonui et al. (16) observed that the level of expression of these histone genes was even much higher in stages in the arthropod vector (sporoblast and sporozoite) than in the schizont stage. Our results are also consistent with those obtained in a *T. parva* proteome characterization study, where histone protein family members were highly expressed (48). It was reported that histone modification operates in synteny with transcription factors and is mostly activated during the replicative schizont stage (49, 50). Experiments using apicidin, a histone deacetylase inhibitor, were shown to alter parasite differentiation status, leading to the conclusion that epigenetic control plays a key role in apicomplexan differentiation steps (51). This observation is consistent with our results, which show histone-encoding genes to be among the most highly differentially expressed genes across the parasite life cycle studied. Genes encoding known proteins such as cysteine proteinase (TP03_0281) and heat shock protein 70 (TP02_0148) were also found very highly expressed in the piroplasm, with, respectively, 22,581 and 15,381 TPM. TP03_0281 protein contains a TMD and secretion activity. This is not unexpected, as these genes encode enzymes that degrade protein and could be required for morphological events or degradation of host cell proteins (52).

All known antigens (45, 53, 54) were expressed at each developmental stage (Table 4), except for p67; this protein is known to be a sporozoite surface protein and only present in the sporozoite infection stage, although the gene was shown recently to be highly expressed by the sporoblast stage (16). We also observed that the expression of known antigens is not conserved across the stages (Figure 5). We included in our analysis the two mammalian stages (schizont and piroplasm) of the parasite and the two vector stages (sporoblast and sporozoite) from our previous work (16), to identify potential new vaccine antigens as well as novel therapeutic targets among those with a similar expression pattern to *T. parva* known antigens. Unsupervised clustering was used to cluster the expressed genes in the parasite life stages. This method has been used in related studies on gene expression data to identify markers associated with virulence, disease pathology that could be used for reverse vaccinology and therapeutic target identification (14, 15, 55). Many co-expressed genes are often co-regulated and have similar expression profiles. Thus, genes with similar expression to the known antigen genes could aid in identifying novel protein targets to improve control strategies against ECF. Since *T. parva* is an intracellular parasite, we prioritized identification genes coding for proteins that are secreted by the parasite (containing an SP) or that are located on the surface of the parasite, i.e., containing TMDs or a predicted GPI anchor, and those that contain predicted antibody target epitopes (Table 5) (15, 56). Combining these characteristics and using the p67 and the diagnostic antigen PIM as benchmarks, we identified a number of potential candidate vaccine antigens [some of which were also identified in a previous study (16)] that are likely to be target of protective immune responses during ECF. In that regard, the ELISA results for TP04_0076 and TP04_0640 showed that these proteins induced antibody reactions in naturally *T. parva-*infected cattle. Particularly, TP04_0076 antigen could induce antibody reactions in some infected cattle at greater levels than the benchmark diagnostic antigen PIM. This is consistent with a previous report that ITM-immunized animals recognized recombinant TP04_0076 (57). A BLASTN revealed a homology to the conserved *Theileria annulata* gene XM_947675.1 (86.12% sequence identity, E-value of 8e−55), to the immunodominant *Plasmodium falciparum* UB05 gene (KF875450.1; 100% sequence identity, E-value of 9e−149), and to the conserved *Theileria orientalis* strain Shintoku gene XM_009694457.1 (80% sequence identity, E-value of 4e−04). It is reported that the TP04_0076 antigen was better at detecting antigen-specific antibodies in the plasma of human subjects with malaria compared to *Plasmodium* homolog UB05 when tested by ELISA (57). TP04_0640 was also found similar to the meiotic upregulated gene MUG84 of yeast endoplasmic reticulum membrane encoding for phosphatidylinositol N-acetylglucosaminyltransferase subunit P (PIG-P) (16). This protein is an enzyme involved in GPI anchor biosynthesis. However, further investigation is necessary to determine if these antigens can be protective against ECF and to evaluate if they can prevent sporozoite infection of cattle or block piroplasm transmission to ticks.

This study expanded our knowledge of *T. parva* genes involved in biological pathways, as this information is limited compared to other apicomplexan parasites (58, 59). We identified transcription factors that were upregulated in the piroplasm including genes involved in nucleic acid and ion binding. A number of hypothetical proteins were also identified as potential SNAREs involved in intracellular vesicular transport. Several of these potential SNARE proteins identified have a TMD and at least a 60-amino-acid-long coiled-coil region as described previously (60). The SNARE interactions shown in Figure 4E involved the plasma membrane, the Golgi body, and the endoplasmic reticulum–related transport pathways. These genes could be investigated for their specific interaction and roles in the development of *T. parva*.

This study shed more light upon gene expression variation as the apicomplexan protozoa *T. parva* develops through its life cycle stages in the tick vector and bovine host, resulting in the establishment of ECF. In addition, bioinformatic analysis of transcriptomics data identified potential candidate vaccine antigens yet to be evaluated for their immunogenicity and potential to induce either humoral or cellular immunity.

## Data Availability Statement

The nucleotide sequence data sets generated in this study can be found in the NCBI database under the accession number: PRJNA604662; https://www.ncbi.nlm.nih.gov/sra/PRJNA604662.

## Ethics Statement

The animal study was reviewed and approved by the ILRI's Institutional Animal Care and Use Committee (IACUC). The study reported here was carried out in strict accordance with the recommendations in the standard operating procedures of the ILRI IACUC and adequate consideration of the 3R's (Replacement of animal with non-animal techniques, Reduction in the number of animals used, and Refinement of techniques and procedures that reduce pain and distress). The ILRI's Experimental Animal Request Form and Protocol for blood collection was approved by the ILRI IACUC (IACUC ref no. 2006.9, IACUC ref 2006.10, IACUC ref 2007.10 and IACUC-RC2015-23).

## Author Contributions

Sample collection and RNA extraction by RP, KA, and EM. ELISA by KA, EM, and RP. Data analysis by KA, JJ, and RP. Initial draft written by KA. Methods validation by RP, JS, and KA. Supervision by RP and JO. Final manuscript revision by KA, CT, JO, AD, JS, and RP. All authors contributed to the writing and manuscript editing.

## Conflict of Interest

The authors declare that the research was conducted in the absence of any commercial or financial relationships that could be construed as a potential conflict of interest. The reviewer LMF declared a past co-authorship with one of the authors JS to the handling editor.

## Supplementary Material

Supplementary Data File 1 Transcript abundance of each sample replicate.

Supplementary Data File 2 List of the stage-specific genes.

Supplementary Data File 3 List of genes associated to the GO term and KEGG term.

Supplementary Data File 4 Genes with similar expression pattern to the known antigens.

Supplementary Data File 5 Candidate vaccine proteins predicted to be localized in the parasite plasma membrane.

Supplementary Data File 6 ELISA OD reading of each epitope sera and the positive and negative control sera.
