# Supplementary material for: Comparative Transcriptomics of the Bovine Apicomplexan Parasite Theileria parva Developmental Stages Reveals Massive Gene Expression Variation and Potential Vaccine Antigens
Source: Front Vet Sci. 2020 Jun 9;7:287. doi: 10.3389/fvets.2020.00287 (PMC7296165; doi:10.3389/fvets.2020.00287)
Supplement: Supplementary Data File 2 — List of the stage-specific genes. [file Data_Sheet_2.docx]

**Supplementary Data File 2 |** Stage specific expressed genes in the two parasite stages

| GenBank Acc. | Gene_ ID | TPM values | | Domains | Product name |
| --- | --- | --- | --- | --- | --- |
|  |  | **Schizont** | **Piroplasm** |  |  |
| XM_758142.1 | TP03_0217 | 0 | 3318 | SP | Hypothetical protein |
| XM_757627.1 | TP03_0905 | 0 | 924 | 3 TMD | Hypothetical protein |
| XM_758244.1 | TP03_0319 | 0 | 463 | (-) | Hypothetical protein |
| XM_757628.1 | TP03_0906 | 0 | 156 | 5TMD | Hypothetical protein |
| XM_760967.1 | TP01_0540 | 0 | 130 | (-) | Hypothetical protein |
| XM_757644.1 | TP03_0922 | 0 | 117 | 5TMD | Hypothetical protein |
| XM_757632.1 | TP03_0910 | 0 | 109 | 4 TMD | Hypothetical protein |
| XM_757622.1 | TP03_0924 | 0 | 76 | 6TMD | Hypothetical protein |
| XM_757633.1 | TP03_0911 | 0 | 72 | 5TMD | Hypothetical protein |
| XM_757626.1 | TP03_0928 | 0 | 66 | 5TMD | Hypothetical protein |
| XM_757636.1 | TP03_0914 | 0 | 66 | 5TMD | Hypothetical protein |
| XM_757634.1 | TP03_0912 | 0 | 63 | 9TMD | Hypothetical protein |
| XM_760347.1 | TP02_0872 | 0 | 62 | SP | Hypothetical protein |
| XM_760435.1 | TP01_0001 | 0 | 55 | (-) | Hypothetical telomeric SfiI 20 protein 1 |
| XM_757630.1 | TP03_0908 | 0 | 54 | 4 TMD | Hypothetical protein |
| XM_757630.1 | TP03_0908 | 0 | 54 | 4TMD | Hypothetical protein |
| XM_758678.1 | TP04_0136 | 0 | 52 | (-) | Hypothetical protein |
| XM_757646.1 | TP03_0615 | 0 | 47 | 4TMD | Hypothetical protein |
| XM_757643.1 | TP03_0921 | 0 | 46 | 4TMD | Hypothetical protein |
| XM_761572.1 | TP01_1144 | 0 | 43 | SP, 1TMD | Hypothetical protein |
| XM_757647.1 | TP03_0616 | 0 | 41 | (-) | Hypothetical protein |
| XM_760770.1 | TP01_0336 | 0 | 39 | SP | Hypothetical protein |
| XM_760436.1 | TP01_0002 | 0 | 38 | SP, 1TMD | Hypothetical telomeric SfiI 20 protein 2 |
| XM_758627.1 | TP04_0085 | 0 | 37 | (-) | Hypothetical protein |
| XM_757641.1 | TP03_0919 | 0 | 35 | 8TMD | Hypothetical protein |
| XM_757668.1 | TP03_0637 | 0 | 33 | (-) | Hypothetical protein |
| XM_761112.1 | TP01_0684 | 0 | 30 | (-) | Hypothetical protein |
| XM_760987.1 | TP01_0561 | 0 | 29 | (-) | Hypothetical protein |
| XM_759132.1 | TP04_0590 | 0 | 29 | 10TMD | Hypothetical protein |
| XM_759898.1 | TP02_0425 | 0 | 27 | SP | Hypothetical protein |
| XM_760612.1 | TP01_0178 | 0 | 26 | SP | Hypothetical protein |
| XM_758988.1 | TP04_0446 | 0 | 25 | (-) | Cell division control protein 2, putative |
| XM_757645.1 | TP03_0614 | 0 | 24 | 3 TMD | Hypothetical protein |
| XM_757624.1 | TP03_0926 | 0 | 23 | 8TMD | Hypothetical protein |
| XM_761485.1 | TP01_1057 | 0 | 23 | SP | Hypothetical protein |
| XM_761075.1 | TP01_0647 | 0 | 21 | (-) | Hypothetical protein |
| XM_760352.1 | TP02_0877 | 0 | 21 | (-) | Hypothetical protein |
| XM_760692.1 | TP01_0258 | 0 | 20 | (-) | Hypothetical protein |
| XM_758891.1 | TP04_0349 | 0 | 20 | (-) | Hypothetical protein |
| XM_758154.1 | TP03_0229 | 0 | 20 | (-) | Hypothetical protein |
| XM_760308.1 | TP02_0833 | 0 | 19 | (-) | Protein kinase domain-containing protein |
| XM_760966.1 | TP01_0539 | 0 | 19 | SP | Hypothetical protein |
| XM_758639.1 | TP04_0097 | 0 | 18 | SP | Hypothetical protein |
| XM_760043.1 | TP02_0570 | 0 | 15 | (-) | Hypothetical protein |
| XM_758397.1 | TP03_0903 | 0 | 15 | (-) | Hypothetical protein |
| XM_761108.1 | TP01_0680 | 0 | 15 | SP | Hypothetical protein |
| XM_761402.1 | TP01_0974 | 0 | 15 | 4TMD | Hypothetical protein |
| XM_759386.1 | TP04_0842 | 0 | 15 | (-) | Hypothetical protein |
| XM_758193.1 | TP03_0268 | 0 | 14 | SP | 6-Cys domain-containing protein |
| XM_757666.1 | TP03_0635 | 0 | 14 | SP | Hypothetical protein |
| XM_758167.1 | TP03_0242 | 0 | 14 | (-) | Hypothetical protein |
| XM_760276.1 | TP02_0802 | 0 | 14 | (-) | Hypothetical protein |
| XM_761247.1 | TP01_0819 | 0 | 14 | (-) | Hypothetical protein |
| XM_759250.1 | TP04_0707 | 0 | 14 | 8TMD | Hypothetical protein |
| XM_758987.1 | TP04_0445 | 0 | 13 | (-) | Cyclin N-terminal domain-containing protein |
| XM_761006.1 | TP01_0578 | 0 | 13 | (-) | Hypothetical protein |
| XM_760871.1 | TP01_0437 | 0 | 13 | 7TMD | Hypothetical protein |
| XM_761050.1 | TP01_0622 | 0 | 12 | (-) | Hypothetical protein |
| XM_758006.1 | TP03_0080 | 0 | 10 | (-) | Hypothetical protein |
| XM_760291.1 | TP02_0816 | 0 | 10 | (-) | Hypothetical protein |
| XM_758416.1 | TP03_0482 | 0 | 10 | 1TMD | Hypothetical protein |
| XM_758028.1 | TP03_0103 | 0 | 10 | (-) | Hypothetical protein |
| XM_760340.1 | TP02_0865 | 0 | 10 | (-) | Hypothetical protein |
| XM_758997.1 | TP04_0455 | 0 | 10 | 10TMD | Hypothetical protein |
| XM_758006.1 | TP03_0080 | 0 | 10 | (-) | Hypothetical protein |
| XM_760102.1 | TP02_0629 | 0 | 9 | (-) | 6-Cys domain-containing protein |
| XM_760768.1 | TP01_0334 | 0 | 9 | SP | Hypothetical protein |
| XM_758869.1 | TP04_0327 | 0 | 9 | (-) | Hypothetical protein |
| XM_757999.1 | TP03_0073 | 0 | 9 | (-) | Hypothetical protein |
| XM_758950.1 | TP04_0408 | 0 | 8 | (-) | Hypothetical protein |
| XM_757642.1 | TP03_0920 | 0 | 8 | 7TMD | Hypothetical protein |
| XM_757887.1 | TP03_0856 | 0 | 8 | SP | Hypothetical protein |
| XM_758144.1 | TP03_0219 | 0 | 8 | SP | Hypothetical protein |
| XM_760630.1 | TP01_0196 | 0 | 7 | (-) | Hypothetical protein |
| XM_759133.1 | TP04_0591 | 0 | 7 | 1 TMD | Hypothetical protein |
| XM_760869.1 | TP01_0435 | 0 | 7 | (-) | Hypothetical protein |
| XM_758411.1 | TP03_0477 | 0 | 7 | (-) | Hypothetical protein |
| XM_757732.1 | TP03_0701 | 0 | 7 | (-) | Protein kinase domain-containing protein |
| XM_758029.1 | TP03_0104 | 0 | 7 | (-) | Hypothetical protein |
| XM_758172.1 | TP03_0247 | 0 | 7 | 2SP | Hypothetical protein |
| XM_759637.1 | TP02_0166 | 0 | 6 | SP | MACPF domain-containing protein |
| XM_758643.1 | TP04_0101 | 0 | 6 | SP | Hypothetical protein |
| XM_759946.1 | TP02_0473 | 0 | 6 | 1TMD | Hypothetical protein |
| XM_757635.1 | TP03_0913 | 0 | 6 | 5TMD | Hypothetical protein |
| XM_759140.1 | TP04_0598 | 0 | 6 | (-) | Hypothetical protein |
| XM_758629.1 | TP04_0087 | 0 | 6 | (-) | Hypothetical protein |
| XM_758763.1 | TP04_0221 | 0 | 6 | (-) | Hypothetical protein |
| XM_757637.1 | TP04_0247 | 0 | 5.2 | (-) | Hypothetical protein |
| XM_758789.1 | TP04_0247 | 0 | 5 | (-) | Hypothetical protein |
| XM_760262.1 | TP02_0788 | 0 | 5 | (-) | Hypothetical protein |
| XM_760259.1 | TP02_0785 | 0 | 5 | (-) | Hypothetical protein |
| XM_758224.1 | TP03_0299 | 0 | 4 | SP | Hypothetical protein |
| XM_760438.1 | TP01_0004 | 0 | 4 | SP, 1TMD | Hypothetical protein |
| XM_759506.1 | TP02_0030 | 0 | 4 | (-) | Hypothetical protein |
| XM_758645.1 | TP04_0103 | 0 | 4 | SP | Hypothetical protein |
| XM_760968.1 | TP01_0541 | 0 | 4 | (-) | Hypothetical protein |
| XM_758720.1 | TP04_0178 | 0 | 4 | 11TMD | Hypothetical protein |
| XM_760899.1 | TP01_0472 | 0 | 4 | (-) | Hypothetical protein |
| XM_760330.1 | TP02_0855 | 0 | 4 | (-) | Hypothetical protein |
| XM_757707.1 | TP03_0676 | 0 | 4 | 1TMD | Aspartyl protease, putative |
| XM_758288.1 | TP03_0361 | 0 | 4 | (-) | Hypothetical protein |
| XM_758668.1 | TP04_0126 | 0 | 4 | (-) | Hypothetical protein |
| XM_759598.1 | TP02_0122 | 0 | 4 | (-) | Hypothetical protein |
| XM_758645.1 | TP04_0103 | 0 | 4 | SP | Hypothetical protein |
| XM_757947.1 | TP03_0021 | 0 | 3 | 9 TMD | Hypothetical protein |
| XM_759867.1 | TP02_0394 | 0 | 3 | (-) | Hypothetical protein |
| XM_760868.1 | TP01_0434 | 0 | 3 | (-) | Hypothetical protein |
| XM_760341.1 | TP02_0866 | 0 | 3 | (-) | Hypothetical protein |
| XM_760567.1 | TP01_0133 | 0 | 3 | (-) | Hypothetical protein |
| XM_760867.1 | TP01_0433 | 0 | 3 | 9 TMD | Hypothetical protein |
| XM_759385.1 | TP04_0841 | 0 | 3 | (-) | Hypothetical protein |
| XM_760329.1 | TP02_0854 | 0 | 3 | SP | Hypothetical protein |
| XM_761262.1 | TP01_0834 | 0 | 3 | (-) | Hypothetical protein |
| XM_758687.1 | TP04_0145 | 0 | 3 | (-) | Hypothetical protein |
| XM_760889.1 | TP01_0462 | 0 | 3 | (-) | Hypothetical protein |
| XM_759508.1 | TP02_0032 | 0 | 3 | (-) | Hypothetical protein |
| XM_761496.1 | TP01_1068 | 0 | 3 | (-) | Hypothetical protein |
| XM_757613.1 | TP05_0037 | 2060 | 0 | 2TMD | Hypothetical protein |
| XM_757596.1 | TP05_0020 | 1714 | 0 | 3TMD | Hypothetical protein |
| XM_757611.1 | TP05_0035 | 1496 | 0 | TMD | Hypothetical protein |
| XM_757616.1 | TP05_0040 | 1271 | 0 | 2TMD | Hypothetical protein |
| XM_757610.1 | TP05_0034 | 992 | 0 | 2TMD | Hypothetical protein |
| XM_757608.1 | TP05_0032 | 977 | 0 | 1TMD | Hypothetical protein |
| XM_757584.1 | TP05_0008 | 689 | 0 | (-) | Ribosomal protein L14, putative |
| XM_757605.1 | TP05_0029 | 586 | 0 | (-) | DNA-directed RNA polymerase beta' chain |
| XM_757598.1 | TP05_0022 | 567 | 0 | 1 TMD | Hypothetical protein |
| XM_757618.1 | TP05_0042 | 433 | 0 | (-) | [DNA-directed RNA polymerase subunit beta](https://david.ncifcrf.gov/geneReportFull.jsp?rowids=3882263) |
| XM_757599.1 | TP05_0023 | 420 | 0 | (-) | ClpC molecular chaperone, putative |
| XM_757604.1 | TP05_0028 | 418 | 0 | (-) | DNA-directed RNA polymerase subunit beta (PEP) |
| XM_757591.1 | TP05_0015 | 409 | 0 | (-) | 50S ribosomal protein L36, apicoplast |
| XM_760366.1 | TP02_0891 | 320 | 0 | (-) | Hypothetical protein |
| XM_757617.1 | TP05_0041 | 277 | 0 | 1TMD | Hypothetical protein |
| XM_760371.1 | TP02_0896 | 266 | 0 | (-) | Hypothetical protein |
| XM_757620.1 | TP05_0044 | 245 | 0 | (-) | Ribosomal protein S2, putative |
| XM_757614.1 | TP05_0038 | 212 | 0 | 2TMD | Hypothetical protein |
| XM_758948.1 | TP04_0406 | 204 | 0 | SP | Hypothetical protein |
| XM_761242.1 | TP01_0814 | 161 | 0 | (-) | Hypothetical protein |
| XM_757581.1 | TP05_0005 | 159 | 0 | 3TMD | Ribosomal protein S3, putative |
| XM_760428.1 | TP02_0954 | 157 | 0 | SP | Hypothetical protein |
| XM_761466.1 | TP01_1038 | 149 | 0 | (-) | Hypothetical protein |
| XM_761466.1 | TP01_1038 | 149 | 0 | (-) | Hypothetical protein |
| XM_761039.1 | TP01_0611 | 149 | 0 | (-) | Hypothetical protein |
| XM_759435.1 | TP04_0891 | 131 | 0 | (-) | FAS1 domain-containing protein |
| XM_758090.1 | TP03_0165 | 131 | 0 | 9 TMD | ABC transporter, putative |
| XM_757587.1 | TP05_0011 | 130 | 0 | (-) | Ribosomal protein S8 |
| XM_757927.1 | TP03_0002 | 123 | 0 | SP | Hypothetical protein |
| XM_759198.1 | TP04_0655 | 115 | 0 | 10 TMD | Hypothetical protein |
| XM_758557.1 | TP04_0015 | 107 | 0 | SP | Hypothetical protein |
| XM_759471.1 | TP04_0002 | 104 | 0 | SP | Hypothetical protein |
| XM_758558.1 | TP04_0016 | 103 | 0 | SP | Hypothetical protein |
| XM_758797.1 | TP04_0255 | 101 | 0 | (-) | Hypothetical protein |
| XM_757928.1 | TP03_0003 | 94 | 0 | SP | Hypothetical protein |
| XM_757588.1 | TP05_0012 | 93 | 0 | (-) | Ribosomal protein L6, putative |
| XM_757619.1 | TP05_0043 | 90 | 0 | 1TMD | DNA-directed RNA polymerase beta chain |
| XM_759463.1 | TP04_0927 | 87 | 0 | SP | Hypothetical protein |
| XM_759462.1 | TP04_0918 | 81 | 0 | SP | Hypothetical protein |
| XM_761044.1 | TP01_0616 | 76 | 0 | (-) | Tash1 protein, putative |
| XM_759460.1 | TP04_0916 | 71 | 0 | SP | Hypothetical protein |
| XM_759486.1 | TP02_0010 | 68 | 0 | SP | Hypothetical protein |
| XM_759461.1 | TP04_0917 | 62 | 0 | SP | Hypothetical protein |
| XM_761045.1 | TP01_0617 | 60 | 0 | (-) | Tash1 protein, putative |
| XM_761045.1 | TP01_0617 | 60 | 0 | (-) | Tash1 protein, putative |
| XM_757601.1 | TP05_0025 | 57 | 0 | (-) | Hypothetical protein |
| XM_761041.1 | TP01_0613 | 55 | 0 | (-) | Hypothetical protein |
| XM_757921.1 | TP03_0889 | 51 | 0 | (-) | Hypothetical protein |
| XM_759464.1 | TP04_0919 | 47 | 0 | SP | Hypothetical protein |
| XM_760427.1 | TP02_0953 | 46 | 0 | SP, TMD | Hypothetical protein |
| XM_757578.1 | TP05_0002 | 42 | 0 | (-) | Ribosomal protein L4 |
| XM_757578.1 | TP05_0002 | 42 | 0 | (-) | Ribosomal protein L4, putative |
| XM_761032.1 | TP01_0604 | 39 | 0 | SP | Hypothetical protein |
| XM_757607.1 | TP05_0031 | 38 | 0 | 2TMD | Hypothetical protein |
| XM_760431.1 | TP02_0957 | 38 | 0 | (-) | Hypothetical protein |
| XM_761030.1 | TP01_0602 | 38 | 0 | (-) | Hypothetical protein |
| XM_761046.1 | TP01_0618 | 37 | 0 | SP | Tash1 protein, putative |
| XM_759688.1 | TP02_0215 | 36 | 0 | SP | Hypothetical protein |
| XM_757917.1 | TP03_0885 | 35 | 0 | SP | Hypothetical protein |
| XM_759686.1 | TP02_0961 | 34 | 0 | SP | Hypothetical protein |
| XM_759687.1 | TP02_0214 | 34 | 0 | SP | Hypothetical protein |
| XM_760429.1 | TP02_0955 | 32 | 0 | SP | Hypothetical protein |
| XM_761034.1 | TP01_0606 | 31 | 0 | (-) | Hypothetical protein |
| XM_757612.1 | TP05_0036 | 30 | 0 | (-) | Hypothetical protein |
| XM_758818.1 | TP04_0276 | 30 | 0 | (-) | Hypothetical protein |
| XM_761031.1 | TP01_0603 | 28 | 0 | SP | TashAT2 protein, putative |
| XM_757922.1 | TP03_0890 | 28 | 0 | SP | Hypothetical protein |
| XM_759689.1 | TP02_0216 | 26 | 0 | 1TMD | Hypothetical protein |
| XM_761042.1 | TP01_0614 | 22 | 0 | (-) | Tash1 protein, putative |
| XM_760432.1 | TP02_0958 | 20 | 0 | SP | Hypothetical protein |
| XM_759682.1 | TP02_0210 | 20 | 0 | SP | Hypothetical protein |
| XM_757751.1 | TP03_0720 | 20 | 0 | 8TMD | Hypothetical protein |
| XM_760434.1 | TP02_0960 | 19 | 0 | SP | Hypothetical protein |
| XM_757925.1 | TP03_0930 | 19 | 0 | SP | Hypothetical protein |
| XM_760433.1 | TP02_0959 | 19 | 0 | SP | Hypothetical protein |
| XM_758553.1 | TP04_0011 | 19 | 0 | SP | Hypothetical protein |
| XM_757779.1 | TP03_0748 | 18 | 0 | 9 TMD | Hypothetical protein |
| XM_757779.1 | TP03_0748 | 18 | 0 | 9TMD | Hypothetical protein |
| XM_757606.1 | TP05_0030 | 17 | 0 | 2TMD | Hypothetical protein |
| XM_757908.1 | TP03_0876 | 16 | 0 | SP | Hypothetical protein |
| XM_758316.1 | TP03_0389 | 16 | 0 | (-) | Hypothetical protein |
| XM_757609.1 | TP05_0033 | 14 | 0 | 1TMD | Hypothetical protein |
| XM_760443.1 | TP01_0009 | 14 | 0 | SP | Hypothetical protein |
| XM_759747.1 | TP02_0274 | 13 | 0 | (-) | Hypothetical protein |
| XM_758171.1 | TP03_0246 | 13 | 0 | (-) | Hypothetical protein |
| XM_757597.1 | TP05_0021 | 13 | 0 | 3TMD | Hypothetical protein |
| XM_757926.1 | TP03_0001 | 12 | 0 | SP | Hypothetical protein |
| XM_759513.1 | TP02_0037 | 12 | 0 | (-) | Hypothetical protein |
| XM_757903.1 | TP03_0871 | 10 | 0 | SP | Hypothetical protein |
| XM_757916.1 | TP03_0884 | 10 | 0 | SP | Hypothetical protein |
| XM_757910.1 | TP03_0878 | 9 | 0 | SP | Hypothetical protein |
| XM_757594.1 | TP05_0018 | 9 | 0 | (-) | Ribosomal protein S7, putative |
| XM_757577.1 | TP05_0001 | 9 | 0 | (-) | Ribosomal protein S4, putative |
| XM_758498.1 | TP03_0563 | 9 | 0 | 11TMD | Hypothetical protein |
| XM_761068.1 | TP01_0640 | 8 | 0 | (-) | Hypothetical protein |
| XM_760247.1 | TP02_0773 | 8 | 0 | (-) | Hypothetical protein |
| XM_759480.1 | TP02_0004 | 8 | 0 | SP | Hypothetical protein |
| XM_759964.1 | TP02_0491 | 7 | 0 | (-) | Hypothetical protein |
| XM_759470.1 | TP04_0001 | 6 | 0 | SP | Hypothetical protein |
| XM_757640.1 | TP03_0918 | 5 | 0 | 7 TMD | Hypothetical protein |
| XM_757580.1 | TP05_0004 | 5 | 0 | (-) | Ribosomal protein S19, putative |
| XM_757905.1 | TP03_0873 | 5 | 0 | SP | Hypothetical protein |
| XM_760891.1 | TP01_0464 | 4 | 0 | (-) | Hypothetical protein |
| XM_757585.1 | TP05_0009 | 4 | 0 | 2TMD | Hypothetical protein |
| XM_757592.1 | TP05_0016 | 4 | 0 | (-) | Ribosomal protein S11, putative |
| XM_760441.1 | TP01_0007 | 4 | 0 | SP | Hypothetical protein |
| XM_760613.1 | TP01_0179 | 4 | 0 | (-) | Hypothetical protein |
| XM_757901.1 | TP03_0869 | 3 | 0 | (-) | Hypothetical protein |
| XM_759431.1 | TP04_0887 | 3 | 0 | (-) | Hypothetical protein |
| XM_758212.1 | TP03_0287 | 3 | 0 | SP, TMD | Sporozoite P67 surface antigen |
| XM_760000.1 | TP02_0527 | 3 | 0 | (-) | Hypothetical protein |

Expression threshold = TPM >2.2 (Genes with a TPM values less than or equal to 2.2 were considered as not expressed and their TPM values were set to 0). TPM, Transcripts Per Kilobase Million; SP, Signal peptide; TMD, Trans-membrane domain; (-), none.
